# Vasopressin SNP pain factors and stress in sickle cell disease

**DOI:** 10.1371/journal.pone.0224886

**Published:** 2019-11-11

**Authors:** Keesha L. Powell-Roach, Yingwei Yao, Ellie H. Jhun, Ying He, Marie L. Suarez, Miriam O. Ezenwa, Robert E. Molokie, Zaijie Jim Wang, Diana J. Wilkie

**Affiliations:** 1 Department of Community Dentistry and Behavioral Science, College of Dentistry, University of Florida, Gainesville, Florida, United States of America; 2 Center for Palliative Care Research and Education, University of Florida College of Nursing, Gainesville, Florida, United States of America; 3 Department of Biobehavioral Nursing Science, University of Florida College of Nursing, Gainesville, Florida, United States of America; 4 Department of Biobehavioral Health Sciences, University of Illinois at Chicago College of Nursing, Chicago, Illinois, United States of America; 5 Committee on Clinical Pharmacology and Pharmacogenetics, University of Chicago, Chicago, Illinois, United States of America; 6 Department of Biopharmaceutical Sciences, University of Illinois at Chicago, Chicago, Illinois, United States of America; 7 Cancer Center, University of Illinois at Chicago, Chicago, Illinois, United States of America; 8 Division of Hematology/Oncology, University of Illinois at Chicago College of Medicine, Chicago, Illinois, United States of America; 9 Jessie Brown Veteran’s Administration Medical Center, Chicago, Illinois, United States of America; University of Mississippi Medical Center, UNITED STATES

## Abstract

**Purpose:**

Frequencies of single nucleotide polymorphisms (SNPs) from pain related candidate genes are available for individuals with sickle cell disease (SCD). One of those genes, the arginine vasopressin receptor 1A gene (AVPR1A) and one of its SNPs, rs10877969, has been associated with pain and disability in other pain populations. In patients with SCD, clinical factors such as pain and stress have been associated with increased health care utilization, but it is not known if the presence of the AVPR1A SNP plays a role in this observation. The study purpose was to explore the relationships between rs10877969 and self-reported pain, stress, and acute care utilization events among individuals with SCD.

**Methods:**

In a cross-sectional investigation of outpatients with SCD, participants completed PAIN*Report*It^®^, a computerized pain measure, to describe their pain experience and contributed blood or saliva samples for genetic analysis. We extracted emergency department and acute care utilization from medical records.

**Results:**

The SNP genotype frequencies (%) for this sample were CC 30 (28%), CT 44 (41%), TT 33 (31%). Acute care utilization and stress as an aggravator of pain were significantly associated with the rs10877969 genotype (*p* = .02 and *p* = .002, respectively). The CT genotype had the highest mean utilization and CC genotype was associated with not citing stress as a pain aggravator. Chronic pain was not associated with rs10877969 (*p* = .41).

**Conclusion:**

This study shows that rs10877969 is related to indicators of stress and acute pain. Further research is recommended with other measures of stress and acute pain.

## Introduction

Clinical factors such as pain and stress have been associated with increased healthcare utilization [1] in patients who have sickle cell disease (SCD), but it is not known if the arginine vasopressin SNP rs10877969 plays a role in this observation. Genetic variability is believed to have a role in the perception of pain in individuals with SCD [2–6]. The differences in pain perception are broad even between individuals with the same SCD genotype [6]. To understand pain in the SCD population, it is important to understand associated genetic factors. Exploring genetic markers, such as SNPs, may be a useful approach to better understand pain [2, 3, 7]. The SNP rs10877969 of the arginine vasopressin receptor gene (AVPR1A), was one of 115 pain associated SNPs analyzed for frequency distribution [8] in a sample of individuals with SCD. In other studies, this SNP was shown to have a three-way interaction with sex, presence of stress at the time of testing, and the perception of pain [9]. Arginine vasopressin is the precursor to nitric oxide production, and nitric oxide has been associated with pain relief [10–12] in patients with SCD [12–18].

Several SNPs (e.g., rs6280, rs920829, rs222747, rs1025928, rs3735942, rs3735943, rs1443952, rs4411417, rs752688, rs10483639, rs3783641, rs8007267) were associated with acute care utilization, a surrogate marker for acute SCD pain, whereas other SNPs (e.g., rs4680 and rs1800587) were associated with chronic SCD pain phenotype.[19, 20]. However, rs10877969 associated pain factors have not yet been explored in patients with SCD, a condition also known for pain and stress [21]. The purpose of this study was to explore the relationships between rs10877969 and acute care utilization events, self-reported pain, and stress among patients with SCD.

SCD is the most severe hemoglobinopathy in the world. It impacts over 100,000 people in the US and millions worldwide [6, 22, 23]. Episodic and severe pain is the hallmark symptom of the disease [6, 23]. Patients suffering from SCD commonly have varying degrees of chronic debilitating pain as well as the episodic acute pain [6, 21, 23] Acute pain is the most common reason for utilization of the emergency department (ED) and hospitalizations [1, 23, 24]. Increased utilization of acute care is associated with early death for individuals ≥20 years of age [1]. In addition, stress had been associated with increased pain in patients with SCD.[21] Furthermore, the Composite Pain Index (CPI), a single score that represents the multiple dimensions of pain, has been shown to predict acute health care utilization [1] and was associated with other SNPs (e.g., rs4680 and rs1800587) in SCD [19, 20]. Associations between rs10877969 and CPI, acute care utilization events and stress have not been previously examined in patients with SCD, and will be examined here.

## Material and methods

### Design

This was a secondary data analysis from a cross-sectional comparative investigation. The study was approved by the Institutional Review Boards at the University of Illinois at Chicago and the University of Florida.

### Sample

Participants were recruited from the University of Illinois Hospital and Health System (UI) Sickle Cell Clinic. Eligibility criteria included: being a patient with a SCD diagnosis who is scheduled for continuing care at the UIC Sickle Cell Clinic, has used opioids for pain crisis, can tolerate the removal of an additional 8.5 ml of blood in addition to their routine labs (not to exceed the safety standard for the subject’s age, weight and health), was age ≥ 18 years, and able to read and speak English. Exclusion criteria included: unable to physically complete study questionnaire, legally blind, and on a chronic transfusion program.

One hundred-seven African American adults with SCD participated. The sample mean age was 35.2 ± 12.0 years. Nearly all (97%) of the participants were African American and 68% were female. The other details of the demographic characteristics of the sample appear in Table 1.

**Table 1:** Demographics and pain characteristics.

Variable	Category/Statistics	Value
Sex	female	73 (68%)
	male	34 (32%)
Age	Mean (SD), Minimum-Maximum	35.2 (12.0), 19–70
Race	African American	104 (97%)
	White	3 (3%)
Ethnicity	Hispanic/Latina	2 (2%)
	Non- Hispanic	105 (98%)
Sickle Cell Type	SS	85 (79%)
	SC	11 (10%)
	Sβ+	5 (5%)
	Sβ°	5 (5%)
	Sα	1 (1%)
rs10877969 Genotype	C/C	30 (28%)
	C/T	44 (41%)
	T/T	33 (31%)
Stress Spontaneously Mentioned	No Stress	72 (67%)
	Stress	35 (33%)
Composite Pain Index (CPI))	Mean (SD), Minimum-Maximum	41.1 (13.7), 14.8–86.5
Acute Care Utilization	Mean (SD), Minimum-Maximum	4.6 (5.5), 0–38

### Procedures

The participants were approached at the University of Illinois (UI) Hospital sickle cell clinic during routine outpatient visits if they met eligibility criteria. The study procedure was explained to the patients and if they agreed to participate, written informed consent was obtained. The participants completed their initial data collection during a routine clinic visit, at home, or prior to hospital discharge. To capture acute care visits outside of the UI, the participants were followed for 24-months with phone calls scheduled at 2-week intervals. Blood or buccal samples and research measures were obtained after the outpatient visit.

### Measures

#### DNA and genotyping

A salting procedure was used to precipitate and isolate DNA from the participants’ blood samples[25]. QuickGene-mini80 and Quick gene DNA whole blood were used to extract the remainder of the peripheral blood samples. DNA samples were stored at -80°. The SNP rs10877969 was genotyped by the MassARRAY iPLEX Platform (Sequenom, CA, USA).

#### Utilization

Utilization events, defined as the number of admissions to the acute care center or emergency department for pain control over a 12 month period, [1] served as a marker for acute pain in SCD. As reported elsewhere, acute care visits were captured via monitoring of the UI electronic admission records [26]. Bi-weekly phone calls were made to the participants to document acute visits that had occurred at other facilities [1, 26]. One individual abstracted the data from the electronic record. A random subset of 20% of the data was independently abstracted by two additional individuals who did not know the aim of the study. The interrater reliability (IRR) of the data abstraction was 92%, which provided evidence of minimal bias [26].

#### Pain

The PAIN*Report*It^®^ software program (Nursing Consult LLC, Seattle, WA) was used to collect pain and demographic data, typically at a routine outpatient visit, as an indication on the patient’s chronic pain. PAIN*Report*It^®^ is a well-validated, modified computerized version of the McGill Pain Questionnaire (MPQ) that measures pain intensity, location, quality, and pattern [1, 27, 28]. This is of value because validity and reliability for the MPQ has been backed by decades of research and in many populations, cultures, and languages, by many investigators [1, 27, 28]. PAIN*Report*It^®^ requires little to no previous computer experience [1, 29]. Using PAIN*Report*It^®^ data, a single Composite Pain Index [30] score was calculated for each participant from the average pain intensity (current, least and worst), number of pain sites, pain pattern, and Pain Rating Index-Total scores.[28] The proportional scores were averaged to generate the CPI score (range 0–100). The CPI is a new way of conceptualizing and scoring the MPQ as a patient-reported outcome using a single score [30] and when collected at a routine outpatient clinic visit, has been shown to be a strong predictor of acute care utilization over the subsequent year in adults with SCD and replicated over a second subsequent year [1].

#### Stress

**Fig 1:**
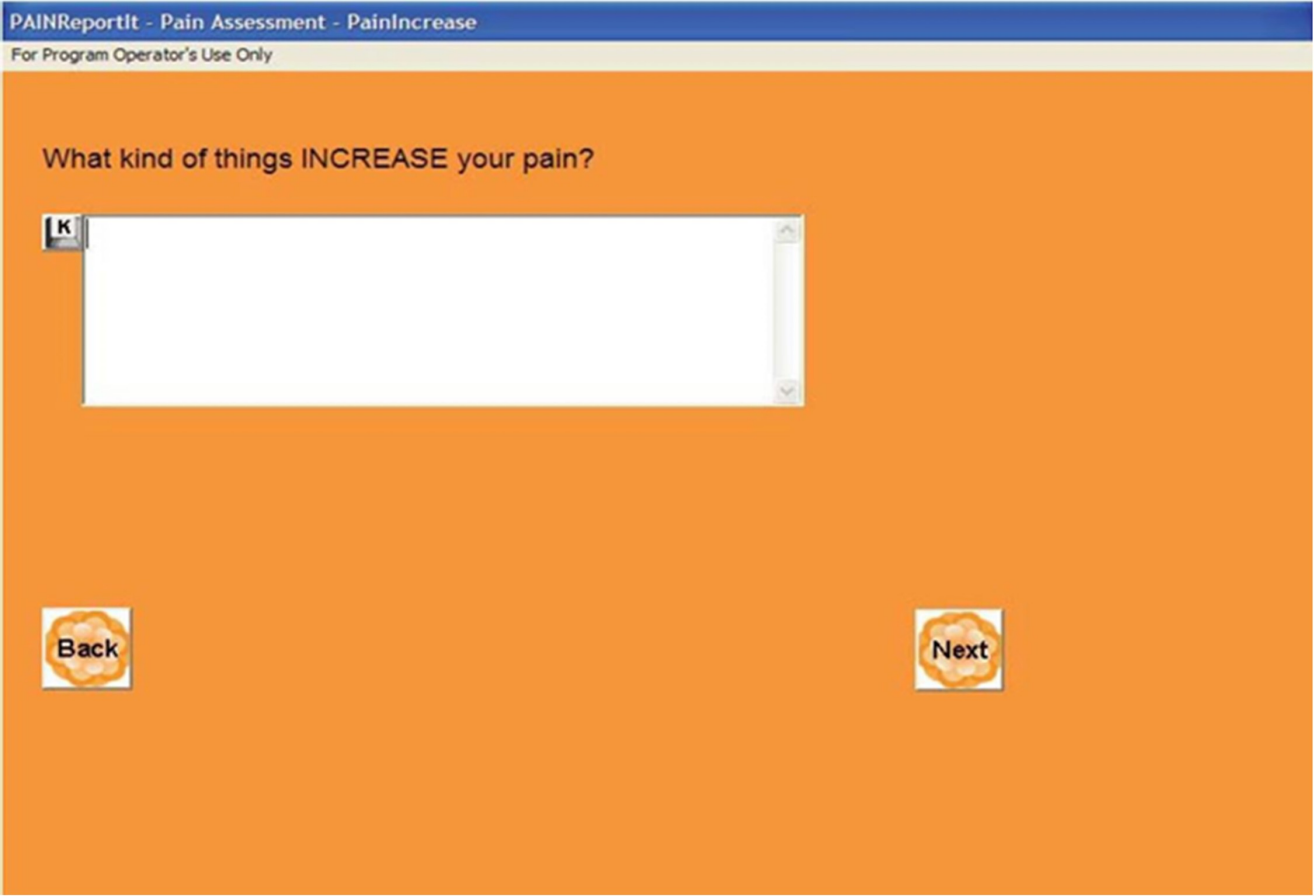
PAIN*Report*It item used to identify spontaneous report of stress as an aggravator of pain.

We examined patients’ reports of stress by reviewing free text descriptions of pain aggravators from PAIN*Report*It^®^ (Fig 1). This information was coded independently by two researchers to create an indicator of stress (stress or no stress mentioned as an aggravator of pain) for use in the data analysis. No self-report of stress as an aggravator of pain was coded 0, and spontaneous reports of stress as an aggravator of pain was coded 1. The researchers used a consensus process to determine the code when their coding was discrepant, which produced 100% agreement.

### Analysis

Using R statistical software, the data were analyzed for descriptive and inferential statistics. Statistical significance was set at less than .05. We examined the association between the genotypes of AVPR1A SNP rs10877969 and several phenotypic measures. For each measure, we compared a model including the genotypes as a predictor with a reduced model without the genotype predictor using a likelihood ratio test. Quasi-Poisson, linear, and binary logistic regressions were used to model utilization, CPI, and citing stress as a factor, respectively.

## Results

Genotyping of AVPR1A SNP rs10877969 demonstrated that there were 30 (28%) patients with CC genotype, 44 (41%) patients with CT genotype, and 33 (31%) patients with TT genotype (Table 2).

**Table 2:** Bivariate associations between genotypes and phenotypic variables.

Variable	Genotype			p
	CC (n = 30)	CT (n = 44)	TT (n = 33)	
Utilization mean (SD)	3.9 (3.0)	6.1 (7.5)	3.3 (3.5)	0.02
CPI mean (SD)	43.9 (16.5)	39.8 (12.6)	40.2 (12.2)	0.41
Stress percentage	10%	48%	33%	0.002
**Sex**				0.68
Female	19 (63%)	32 (73%)	22 (67%)	
Male	11 (37%)	12 (27%)	11 (33%)	
**Age** [19–70]	36.2 (11.5)	35.9 (12.4)	33.4 (12.0)	0.57
**Sickle cell status**				0.12
SS	23 (77%)	33 (75%)	29 (88%)	
SC	1 (3%)	8 (18%)	2 (6%)	
Sβ+	2 (7%)	1 (2%)	2 (6%)	
Sβ°	3 (10%)	2 (5%)	0 (0%)	
Sα	1 (3%)	0 (0%)	0 (0%)	

Utilization = Acute Care Utilization; CPI = Composite Pain Index; Stress = Stress Spontaneously Mentioned as Pain Aggravator.

### Utilization

The mean (SD) number of acute care utilization events was 4.6 (5.5) and ranged from 0 to 38 (Table 1). The mean utilization events was 3.9 (3.0) for participants with the CC genotype, 6.1 (7.5) for those with the CT genotype, and 3.3 (3.5) for the TT genotype. The utilization events were significantly associated with rs10877969 genotype (p = .02) (Table 2). The association remained significant when controlling for age, sex, and sickle cell status (p = .01).

### CPI

The mean (SD) CPI for the participants was 41.1 (13.7) and ranged from 14.8 to 86.5 (Table 1). The mean CPI for participants with the CC genotype was 43.9 (16.5), the CT genotype was 39.8 (12.6), and the TT genotype was 40.2 (12.2). The CPI was not significantly associated with rs10877969 genotype (Table 2). Controlling for age, sex, and sickle cell status did not change the finding.

### Stress

**Fig 2:**
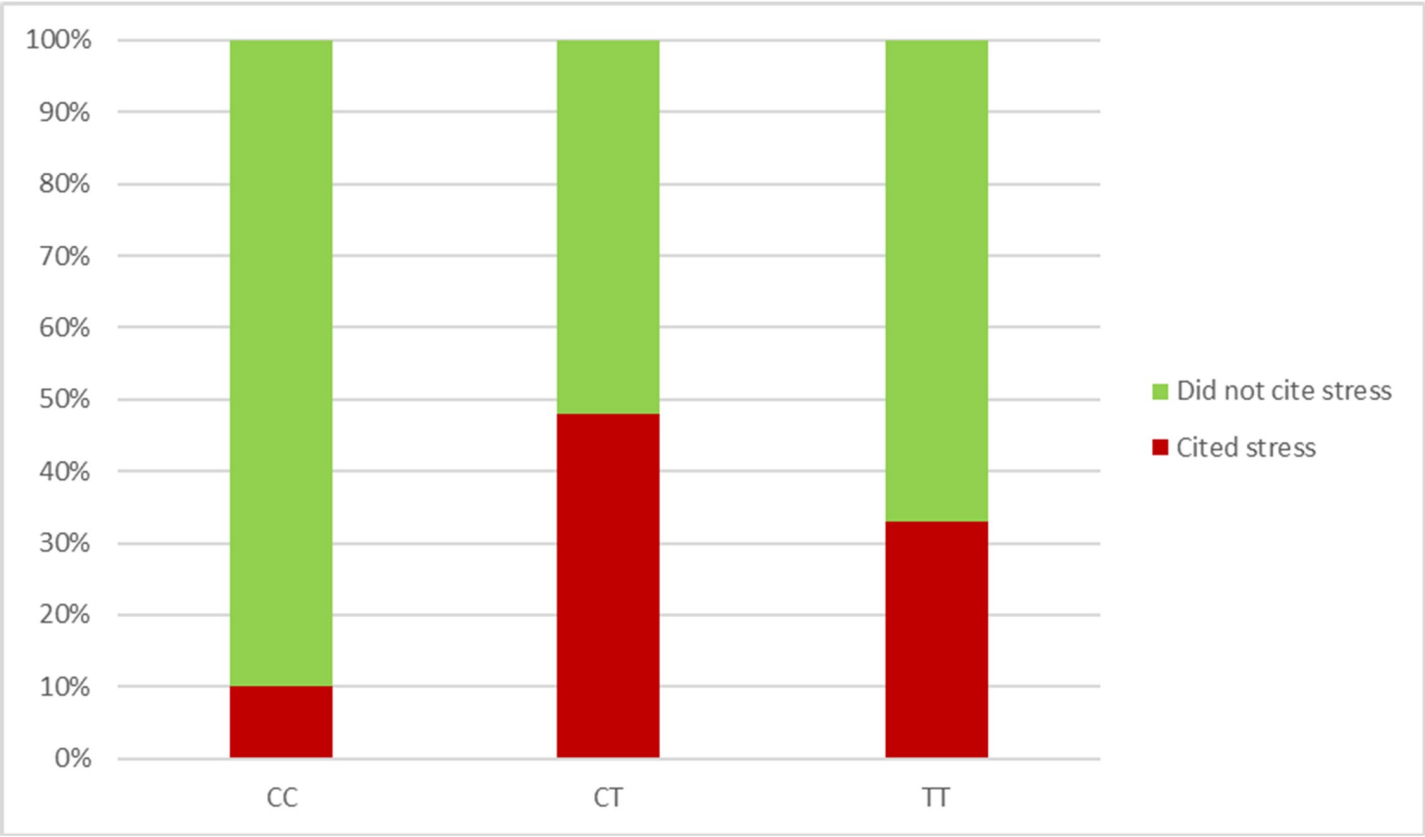
Genotype by stress as a pain aggravator.

Of the 107 participants, 35 (33%) spontaneously reported stress as an aggravator of pain (Table 1). 10% of participants with the CC genotype, 48% with the CT genotype, and 33% with the TT genotype reported stress as an aggravator of pain (Fig 2). Stress cited as a pain aggravator was associated with the rs10877969 genotype (*p* = .002) (Table 2). The association remained significant when controlling for age, sex, and sickle cell status (p = .003).

## Discussion

In a study of 107 adults with SCD, we conducted an association study that revealed three main findings regarding the AVPR1A gene and its SNP, rs10877969. First, individuals with the CT genotype were more likely to have higher acute care utilization events for pain control, the phenotype for acute pain. Second, the CPI, which was the phenotype for chronic pain, was not significantly associated with the SNP rs10877969. Third, when examining the self-reported stress as a factor associated with the pain phenotype, we found that those who were least likely to spontaneously report stress as a pain aggravator had the CC genotype.

Our exploratory findings are different from findings from studies of other pain conditions that included a sample of fewer African Americans [31, 32] than our sample. In an experimental acute pain model of capsaicin induced pain, Mogil et al. found a three-way interaction with stress, sex, and genotype wherein males with stress at the time of testing and CC/CT genotype had low pain compared to the TT genotype [31]. The low pain rating based on genotype was not observed among African American males [31]. In an exercise induced pain model, pain duration was associated with the CC genotype [9]. These findings related to acute pain contrast with our findings from an African American sample where it was the CT genotype that was associated with more frequent acute SCD pain utilization events. Furthermore, those having the CC genotype were less likely to report stress. There was no significant association with chronic pain. Reasons for these differences are unknown, but could be related to differences in pain models, pain and stress measures, or sample characteristics such as age, sex, race/ethnicity, and genetic admixture. Clearly, future research on rs10877969 should include measures of acute pain intensity and perceived stress in larger samples with adequate distribution by age, sex, and ancestry as indicated from ancestral DNA markers rather than self-reported race/ethnicity.

Study of other psychosocial measures and rs10877969 is also warranted. Pain catastrophizing and depressive symptoms were associated with rs10877969 [9]. Previous findings have shown that perceived stress and perceived injustice are associated with severity of SCD pain and should be studied for association with rs10877969 [21, 33]. Additional research is needed to further examine the variations of the SNP rs10877969 by psychosocial, pain, and stress outcomes and demographic characteristics to determine the importance of the SNP for clinical SCD care and for pain research.

### Limitations

A limitation of this study was that we did not formally measure stress, we derived a stress measure from individuals who spontaneously reported stress as an aggravator of pain. The number of participants who would actually report stress as an aggravator for pain may be higher than what we observed in this sample if we had included stress specific self-reported questions. Our eligibility criteria excluded patients who had not had recent acute care utilization and it is unknown if findings would differ had they been included. Sample size was also a limitation as this study was exploratory and conducted with an available sample participating in a large study of SCD pain. Finally, it is unknown if similar findings apply to other diasporas with SCD since our sample did not include individuals from Saudi Arabia, India and other countries with a high prevalence of SCD.

## Conclusions

This is the first study of AVPR1A (rs10877969) among individuals with SCD and showed that individuals with the CT genotype have higher acute care utilization whereas those with CC are less likely to spontaneously report stress as a pain aggravator. SCD is a genetic condition known for pain that is associated with stress and inconsistently associated with sex, two variables that other researchers have found to be associated with this SNP. Further research is warranted on rs10877969 and should include pain, perceived stress, and other psychosocial measures in larger samples with adequate distribution by age, sex, and ancestry.

## Supporting information

S1 Data This is the S1 data used for this manuscript. (XLSX)
